# Karyopherin Alpha2 Is Essential for rRNA Transcription and Protein Synthesis in Proliferative Keratinocytes

**DOI:** 10.1371/journal.pone.0076416

**Published:** 2013-10-03

**Authors:** Noriko Umegaki-Arao, Katsuto Tamai, Keisuke Nimura, Satoshi Serada, Tetsuji Naka, Hajime Nakano, Ichiro Katayama

**Affiliations:** 1 Department of Dermatology, Osaka University Graduate School of Medicine, Osaka, Japan; 2 Department of Stem Cell Therapy Science, Osaka University Graduate School of Medicine, Osaka, Japan; 3 Division of Gene Therapy Science, Osaka University Graduate School of Medicine, Osaka, Japan; 4 National Institute of Biomedical Innovation Laboratory for Immune Signal, Osaka, Japan; 5 Department of Dermatology, Hirosaki University School of Medicine, Hirosaki, Japan; University of Tennessee, United States of America

## Abstract

Karyopherin proteins mediate nucleocytoplasmic trafficking and are critical for protein and RNA subcellular localization. Recent studies suggest KPNA2 expression is induced in tumor cells and is strongly associated with prognosis, although the precise roles and mechanisms of KPNA2 overexpression in proliferative disorders have not been defined. We found that KPNA2 expression is induced in various proliferative disorders of the skin such as psoriasis, Bowen’s disease, actinic keratosis, squamous cell carcinoma, Paget’s disease, Merkel cell carcinoma, and mycosis fungoides. siRNA-mediated KPNA suppression revealed that KPNA2 is essential for significant suppression of HaCaT proliferation under starvation conditions. Ribosomal RNA transcription and protein synthesis were suppressed by starvation combined with knockdown of KPNA (including KPNA2) expression. KPNA2 localized to the nucleolus and interacted with proteins associated with mRNA processing, ribonucleoprotein complex biogenesis, chromatin modification, and transcription, as demonstrated by tandem affinity purification and mass spectrometry. KPNA2 may be an important promoter of ribosomal RNA and protein synthesis in tumor cells.

## Introduction

Recent studies have defined the molecular mechanisms of nucleocytoplasmic signal transduction by karyopherins (KPNs), which function as receptors for various intracellular molecules and mediate nuclear import and export during interphase. In humans, the karyopherin alpha (KPNA) family consists of at least 7 family members, all of which interact with karyopherin beta (KPNB) 1 and transport various proteins and RNAs through the nuclear pores in an energy-dependent manner [1]–[3]. Various extracellular environmental changes activate intracellular signaling cascades by which cells exchange activated signaling molecules between the nucleus and cytoplasm via the KPN-mediated machinery to regulate proliferation and differentiation status [2], [4]–[6]. KPNA2 expression in human epidermal keratinocytes, but not in human dermal fibroblasts, is differentially regulated by transforming growth factor (TGF)-β_1_ and interferon (IFN)-γ, both of which are established modulators of epidermal proliferation and differentiation [4]. KPNA2 also mediates the translocation of epidermal differentiation-inducing signals into the nucleus by recruiting transcription factors such as interferon regulatory factor-1 (IRF-1), thereby inducing IFN-γ-mediated epidermal differentiation [4]. Karyopherin alphas also mediate mitotic spindle assembly [7]–[9] and nuclear membrane formation [10]. KPNB1 is also a global regulator of mitotic spindle assembly, centrosome dynamics, nuclear membrane formation, and nuclear pore complex assembly [11], [12]. Recent studies have revealed that KPNs including KPNA2 are overexpressed in various kinds of tumors such as breast cancer, cervical cancer, non-small cell lung cancer, prostate cancer, and primary cutaneous melanoma, and that expression levels in these tumors are closely associated with prognosis [13]–[18]. Nevertheless, the precise roles and mechanisms of KPN overexpression in proliferative disorders have not been defined.

The rate of cell growth and proliferation is proportional to the rate of protein synthesis, which is tightly linked to ribosome biologics [19], [20]. RNA synthesis and ribosome construction occur in the nucleolus and their control is important for regulating protein synthesis; however, the precise mechanisms and roles of karyopherins in regulating rRNA and protein synthesis remain unclear.

We report KPNA2 induction in proliferation disorders regardless of malignancy, and suggest KPNA2 regulates rRNA transcription and general protein synthesis in the nucleolus to maintain proliferation.

## Materials and Methods

### Skin Samples

Written informed consent was obtained from all patients, and the study protocol was approved by Medical Ethics Committee of Osaka University.

### Cell Culture

HaCaT cells, an immortalized, nontumorigenic keratinocyte cell line, were cultured in Dulbecco’s modified Eagle’s medium (DMEM; Nacalai Tesque) containing 10% fetal bovine serum (FBS) at 37°C under 5% CO_2_-95% air.

### RNA Purification and Reverse Transcription-quantitative Polymerase Chain Reaction

Total RNA was isolated from HaCaT cells with an RNA isolation kit (Qiagen) and reverse transcribed with SuperScript III reverse transcriptase (Invitrogen). Expression of pre-rRNA was determined by using Power SYBR green PCR Master Mix (Applied Biosystems) according to the manufacturer’s protocol. β-Actin was used to normalize target gene expression. PCR amplification was performed with 5′-ATCGTCCACCGCAAATGCTTCTA-3′ and 5′-AGCCATGCCAATCTCATCTTGTT-3′ for β-actin and 5′-GAACGGTGGTGTGTCGTTC-3′ and 5′-GCGTCTCGTCTCGTCTCACT-3′ for pre-rRNA [21]. PCR cycling conditions were 40 cycles of denaturing at 92°C for 15 sec and annealing at 60°C for 60 sec on an ABI Prism 7000 sequence detection system (Applied Biosystems).

### Small Interfering RNA and Plasmid DNA Transfection

Small interfering RNAs (siRNAs) specific for KPNA1, 2, 3, and 4 and the control stealth siRNA were obtained from Invitrogen. Cells (1.5×10^6^) were transfected with 100 ng siRNAs mixture using the Neon transfection system (Invitrogen). We performed the knockdown studies with each siRNA, which ensured more than 50–70% suppression of KPNA2 mRNA and protein.

### MTS

#### [3-(4,5-dimethylthiazol-2-yl)-5(3-carboxymethoxyphenyl)-2-(4-sulfophenyl)-2H-tetrazolium] assay

HaCaT cells induced with each siRNA were seeded at their optimal cell density (7×10^4^ cells/well) in 96-well microtiter plates and incubated to allow cell attachment. After 6 h, cells were incubated with 0.1% FBS DMEM for 24, 48, 72, and 120 h. At the end of each incubation period, cell viability was determined by using the CellTiter 96® AQ_ueous_ Non-Radioactive Cell Proliferation Assay (Promega) according to the manufacturer’s instructions. Samples were incubated at 37°C in a humidified 5% CO_2_ atmosphere for 1 h. Absorbance was measured at 490 nm using a microplate reader.

### Immunohistochemistry

Slides of skin biopsies in paraffin blocks were stained with hematoxylin and eosin (HE) and anti-human KPNA2 mouse monoclonal antibody (BD Biosciences) (1∶1000).

### Immunofluorescence

HaCaT cells were fixed in 4% formaldehyde in phosphate-buffered saline (PBS) for 40 min. After rinsing twice with PBS, the cells were permeabilized in 0.5% Triton X-100 in PBS for 60 s and blocked with 2% skim milk overnight at 4°C. The cells were incubated with anti-UBF (Santa Cruz) and anti-KPNA2 antibodies for 1 h and stained with Alexa Fluor 546 goat anti-rabbit IgG and Alexa Fluor 488 goat anti-mouse IgG secondary antibodies (1∶1000; Invitrogen A-11035 and A-11029) for 1 h. After washing with PBS, cells were counterstained with 0.5 mg/mL 4′, 6′-diamidino-2-phenylindole (DAPI) and mounted with Vectashield mounting medium (Vector Laboratories). Cells were analyzed using a Radiance 2100 confocal scanning-laser microscope (Bio-Rad) equipped with an Eclipse TE-2000 inverted microscope (Nikon) or a Nikon A1 confocal scanning-laser microscope equipped with a Nikon Eclipse Ti inverted microscope.

### Tandem Affinity Purification (TAP) and Mass Spectrometry

KPNA2 and GFP cDNAs were introduced into pCAGIP-gw-TAP by using Gateway technology (Invitrogen). KPNA2-TAP and GFP-TAP complexes were purified from HaCaT cell extracts using TAP technology [22], [23]. Proteins were separated by SDS-PAGE and stained with the Silver Stain MS Kit (Wako Pure Chemical Industries). Protein bands were excised from the gel and digested with trypsin (Promega) [24]. NanoLC-MS/MS analyses were performed on a LTQ-Orbitrap XL mass spectrometer (Thermo Fisher Scientific) equipped with a nano-ESI source (AMR) and coupled to a Paradigm MG4 pump (Michrom Bioresources) and an autosampler (HTC PAL, CTC Analytics). A spray voltage of 1800 V was applied. The peptide mixture was separated on a MagicC18AQ column (100 µm×150 mm, 3.0 µm particle size, 300 Å, Michrom Bioresources) with a flow rate of 500 nl/min. A linear gradient of 5% to 45% B in 30 min, 45% to 95% B in 0.1 min, and 95% B for 2 min and 5% B was employed (A = 0.1% formic acid in 2% acetonitrile, B = 0.1% formic acid in 90% acetonitrile). Intact peptides were detected in the Orbitrap at 60,000 resolutions. For LC-MS/MS analysis, 6 precursor ions were selected for MS/MS scans in a data-dependent acquisition mode following each full scan (m/z, 350–1500). A lock mass function was used for the LTQ-Orbitrap to obtain constant mass accuracy during gradient analysis.

Peptides and proteins were identified by automated database searching the Swiss-Prot protein database (version 57.14x) with the MASCOT search program (version 1.0; Matrix Science) and a precursor mass tolerance of 10 p.p.m., a fragment ion mass tolerance of 0.8 Da, and strict trypsin specificity, allowing for up to 2 missed cleavages. Carbamidomethylation of cysteine was set as a fixed modification and oxidation of methionines was allowed as a variable modification.

### Metabolic Labeling

HaCaT cells were labeled for 2 h with 100 mCi ^35^S-methionine in methionine-free DMEM (Gibco) supplemented with 10% dialyzed serum. Protein was extracted with TNE buffer containing 50 mM Tris-HCl at pH 7.4, 150 mM NaCl, 2 mM EDTA, and 0.5% NP-40, then resuspended in 1% sodium dodecyl sulfate and boiled for 10 min at 100°C. Radioactivity was measured with a Beckmann Coulter liquid scintillation counter and normalized to protein content.

### Transient Transfection and Luciferase Assay

The human pre-rRNA-luc vector was kindly provided by Dr. Samson Jacob [25]. HaCaT cells induced with each siRNA were seeded in a 12-well plate and transfected with 0.34 µg human-pre-rRNA-luc plasmid and Fugene 6 transfection reagent (Roche). The luciferase reporter assay was performed using a commercial luciferase assay kit (Promega). Data were normalized to the protein concentration.

### Statistical Analysis

All data and results were confirmed in at least 3 independent experiments. Statistical significance was determined by one-way analysis of variance (ANOVA).

## Results

### KPNA2 Overexpression in Proliferative Disorders of the Skin

**Figure 1:**
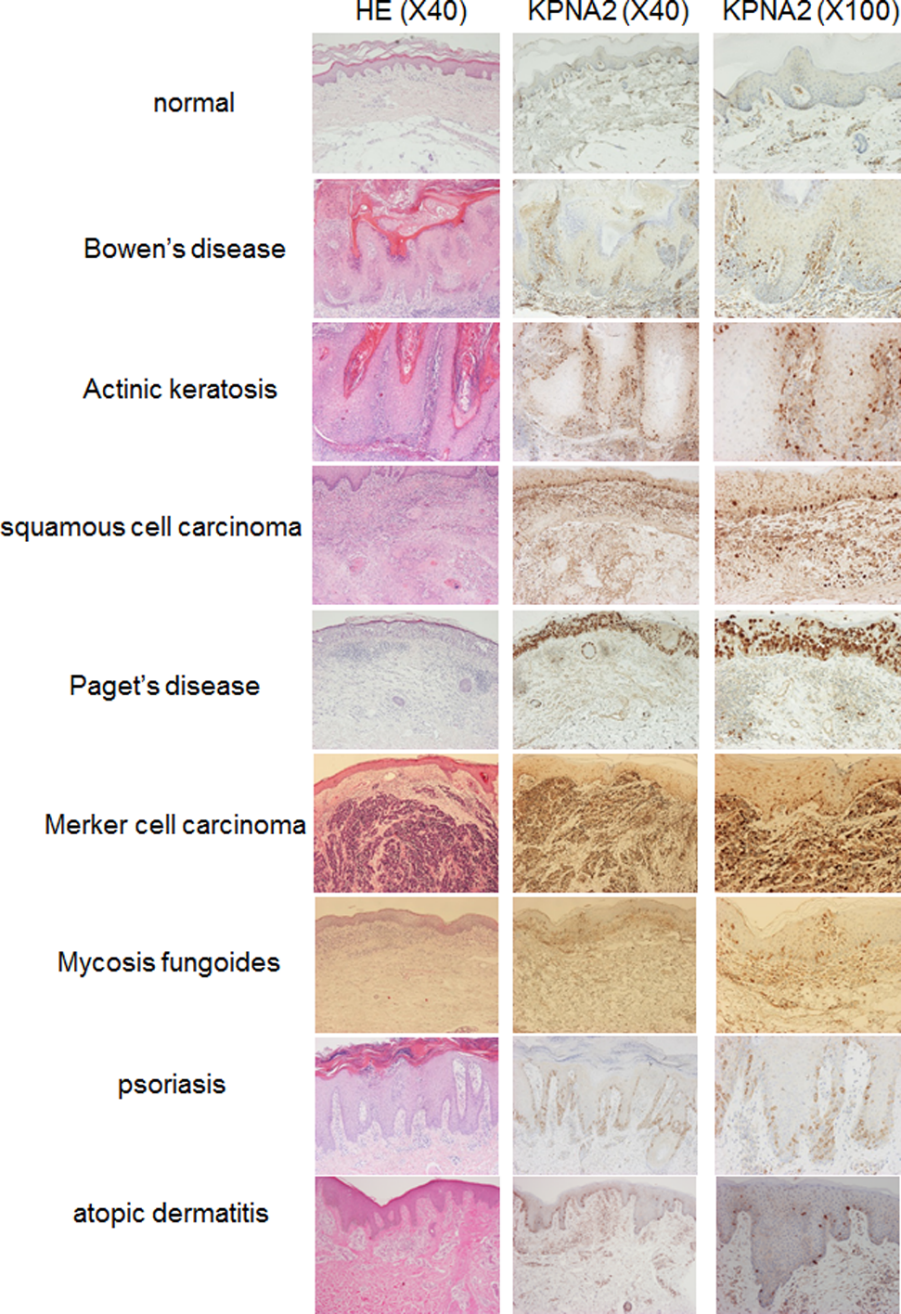
Overexpression of KPNA2 in proliferating cells. Immunohistochemistry showed KPNA2 was uniformly expressed throughout the epidermis in healthy skin, although KPNA2 overexpression was observed in the basal layer in psoriasis. In contrast, very few cells exhibited KPNA2 staining in the basal cells of atopic dermatitis. KPNA2 overexpression was observed in the tumor cells of Bowen’s disease, actinic keratosis, squamous cell carcinoma, Paget’s disease, Merkel cell carcinoma, and mycosis fungoides.

To investigate KPNA2 expression in various epidermal-proliferative disorders of the skin, immunohistochemical staining of KPNA2 was performed on biopsy specimens of epidermal tumors as well as psoriasis and atopic dermatitis, which are inflammatory skin diseases with higher and lower epidermal proliferation, respectively. KPNA2 staining was faint and homogeneous without significant nuclear accumulation in healthy epidermis. In contrast, there was marked KPNA2 staining in the nuclei and cytoplasm of malignant cells in several skin tumors with different prognoses including Bowen’s disease, actinic keratosis, squamous cell carcinoma (SCC), Paget’s disease, Merkel cell carcinoma, and Mycosis fungoides. In malignant cells of SCC *in situ* such as Bowen’s disease and actinic keratosis as with well prognosis, KPNA2 expressed predominantly in the basal layer. In contrast, established SCC showed rather intense and diffuse expression of KPNA2 in the malignant cells. Non-squamous cell malignant tumors of the skin including Paget’s disease, Merkel cell carcinoma, and mycosis fungoides also showed diffuse, intense staining of KPNA2, indicating significantly higher expression in skin malignancy. Marked staining of KPNA2 was also observed in psoriatic skin, but was limited to the cytoplasm of basal layer keratinocytes. In contrast, very few but significant numbers of KPNA2-positive keratinocytes were observed in the basal lesions of atopic dermatitis, particularly in the inflamed proliferating lesions **(** **Figure 1** **)**.

### Contribution of KPNA2 and other KPNAs to Keratinocyte Cell Growth

**Figure 2:**
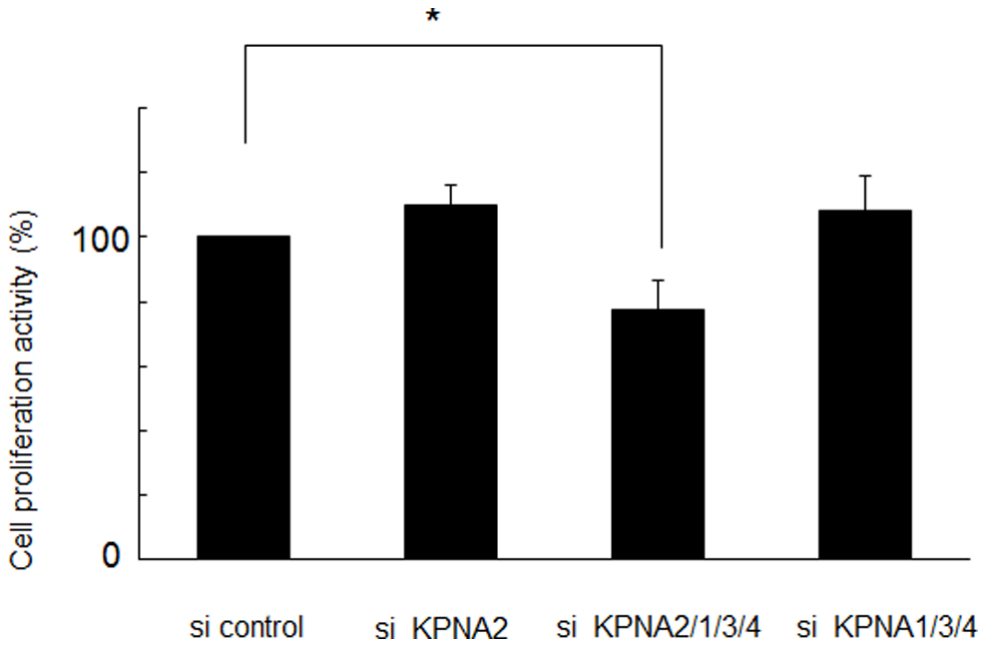
Suppression of cell growth by combined KPNA knockdown. Under starvation conditions (0.1% FBS), siRNA-mediated knockdown of KPNA2, 1, 3, and 4 suppressed cell growth after 120 h (*p<0.05). Only KPNA2 siRNA subtraction produced no change in proliferation.

To assess the role of KPNs in keratinocyte proliferation, HaCaT cell growth in culture was assessed by MTS assay after siRNA-mediated knockdown of KPNs. In culture medium containing 10% FBS, growth was significantly suppressed by KPNB1 knockdown [13]; however, knockdown of other KPNAs produced no significant effect (data not shown). In starved culture medium with 0.1% FBS, HaCaT cell growth was significantly suppressed by siRNA knockdown of KPNA1, 2, 3, and 4, suggesting adequate expression of KPNAs may be required for growth maintenance, especially in starved cells such as cancer cells. About 20% of HaCaT keratinocyte growth was suppressed 120 h after KPNA knockdown. KPNA siRNAs were individually subtracted from the siRNA cocktail to investigate the contribution of each KPNA to growth suppression. Interestingly, only KPNA2 siRNA subtraction resulted in the significant recovery of cell growth up to the control level **(** **Figure 2** **)**, while removal of the other KPNA siRNAs did not affect growth suppression (data not shown). KPNA2 knockdown alone had no significant growth suppression effect, suggesting the other KPNAs are redundant. These data suggest KPNAs complement each other during cell growth, but KPNA2 may be essential for maintaining cell proliferation under starvation conditions.

### Association of KPNA2 with Ribosomal Proteins in the Nucleolus

**Figure 3:**
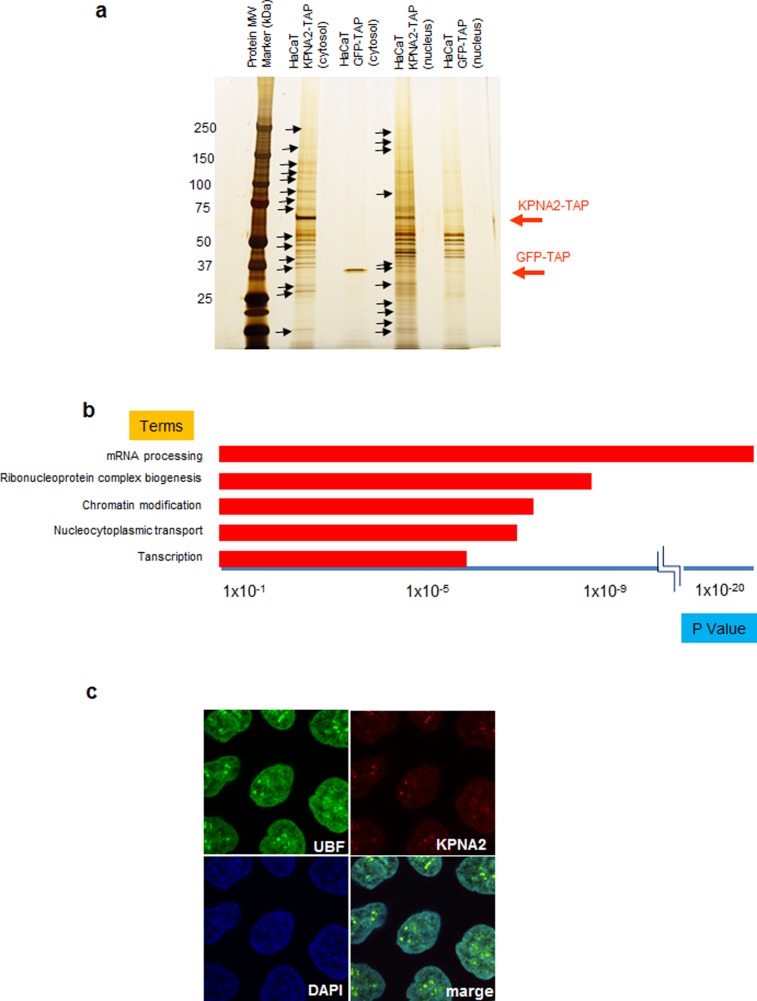
Detection and analysis of proteins that interact with KPNA2 and localization of KPNA2 in the nucleolus. Proteins that interact with KPNA2 in the cytoplasm and nucleus were purified using the TAP method and detected by silver staining. Proteins marked with arrows were analyzed by LC/MS/MS. HaCaT cells expressing GFP-TAP were used to detect nonspecific interactions. **a)** The results of LC/MS/MS were analyzed by pathway analysis using reactome (http://www.reactome.org). The categories of “mRNA processing”, “ribonucleoprotein complex biogenesis”, “chromatin modification,” and “transcription” were the most significantly represented pathways. **b)** Immunohistochemistry revealed KPNA2 co-localization with UBF, a nucleolar marker.

To identify proteins that interact with KPNA2 in HaCaT keratinocytes, we used the TAP method, which enabled us to easily isolate and purify proteins bound to the stably expressed TAP-tagged target recombinant protein [22], [23]. Proteins associated with the KPNA2-TAP complex were isolated from the nuclei and cytoplasm of KPNA2-TAP-expressing HaCaT cells, separated by SDS-PAGE, and silver stained. HaCaT cells expressing GFP-TAP were used as a negative control **(** **Figure 3a** **)**. KPNA2-TAP-associated proteins extracted from the silver-stained gel were identified by mass spectrometry. Numerous proteins were analyzed by reactome (http://www.reactome.org) to investigate their biological relationships. Pathway analysis revealed that the proteins interacting with KPNA2 were associated with mRNA processing, ribonucleoprotein complex biogenesis, chromatin modification, and transcription, all of which are essential for cell activities including cell growth **(** **Figure 3b** **,** **Table 1** **)**. Interestingly, significant numbers of ribosomal proteins were listed as associated with KPNA2. Furthermore, immunofluorescence staining of KPNA2 in cultured HaCaT keratinocytes demonstrated co-localization of KPNA2 with UBF in the nucleoli, suggesting a role of KPNA2 for maintaining rRNA function **(** **Figure 3c** **)**.

**Table 1:** Lists of proteins analyzed by pathway analysis.

mRNA processing			
RALY		RNA-binding protein Raly	
NCBP1		Nuclear cap-binding protein subunit 1	
RNMT		mRNA cap guanine-N7 methyltransferase	
GAR1		H/ACA ribonucleoprotein complex subunit 1	
PABPC4		PABPC4 protein	
MLH1		DNA mismatch repair protein Mlh1	
YBX1		Nuclease-sensitive element-binding protein 1	
SRRT		Serrate RNA effector molecule homolog	
DDX17		Probable ATP-dependent RNA helicase DDX17	
RRP1B		Ribosomal RNA processing protein 1 homolog B	
PCBP1		Poly(rC)-binding protein 1	
PCBP2		Poly(rC)-binding protein 2	
SFRS9		Splicing factor, arginine/serine-rich 9	
PABPC1		Polyadenylate-binding protein 1	
NSUN2		tRNA (cytosine-5-)-methyltransferase NSUN2	
KRR1		KRR1 small subunit processome component homolog	
DHX9		ATP-dependent RNA helicase A	
RRP1		Ribosomal RNA processing protein 1 homolog B	
DDX1		ATP-dependent RNA helicase DDX1	
HNRNPU		Heterogeneous nuclear ribonucleoprotein U	
TTF2		Transcription termination factor 2	
SFRS3		Splicing factor, arginine/serine-rich 3	
PHAX		Phosphorylated adapter RNA export protein	
NOP2		Putative ribosomal RNA methyltransferase NOP2	
RPS16		RPS16 protein	
SNRNP200		U5 small nuclear ribonucleoprotein 200 kDa helicase	
SYF2		Pre-mRNA-splicing factor SYF2	
NOP56		NOP56 protein	
RBM14		RNA-binding protein 14	
BAT1		Spliceosome RNA helicase BAT1	
ADAR		Double-stranded RNA-specific adenosine deaminase	
KIAA1429		Protein virilizer homolog	
**Ribonucleoprotein complex biogenesis**			
NCBP1			Nuclear cap-binding protein subunit 1
KRR1			KRR1 small subunit processome component homolog
RRP1			Ribosomal RNA processing protein 1 homolog B
GAR1			H/ACA ribonucleoprotein complex subunit 1
NIP7			60 S ribosome subunit biogenesis protein NIP7 homolog
DDX1			ATP-dependent RNA helicase DDX1
PHAX			Phosphorylated adapter RNA export protein
NOP2			Putative ribosomal RNA methyltransferase NOP2
RPS16			RPS16 protein
RRP1B			Ribosomal RNA processing protein 1 homolog B
SNRNP200			U5 small nuclear ribonucleoprotein 200 kDa helicase
SFRS9			Splicing factor, arginine/serine-rich 9
NOP56			NOP56 protein
**Chromatin modification**			
ING5			Inhibitor of growth protein 5
RBBP4			Histone-binding protein RBBP4
RBBP7			Histone-binding protein RBBP7
ARID2			AT-rich interactive domain-containing protein 2
CHD8			Chromodomain-helicase-DNA-binding protein 8
HDAC2			Histone deacetylase 2
HDAC1			Histone deacetylase 1
ASH1L			Probable histone-lysine N-methyltransferase ASH1L
BRDT			Bromodomain testis-specific protein
RBM14			RNA-binding protein 14
BCOR			BCL-6 corepressor
CHD4			Chromodomain-helicase-DNA-binding protein 4
MLL2			Histone-lysine N-methyltransferase MLL2
**Nucleocytoplasmic transport**			
PHAX			Phosphorylated adapter RNA export protein
NCBP1			Nuclear cap-binding protein subunit 1
UPF1			Regulator of nonsense transcripts 1
SET			Protein SET
GLE1			Nucleoporin GLE1
NUPL2			Nucleoporin-like protein 2
TPR			Nucleoprotein
KPNA2			Importin subunit alpha-2
KPNB1			Importin subunit beta-1
BAT1			Spliceosome RNA helicase BAT1
**Transcription**			
ING5	Inhibitor of growth protein 5		
NMI	N-myc-interactor		
FOXK1	Forkhead box protein K1		
FOXM1	Forkhead box protein M1		
CCNT1	Cyclin-T1		
ARID2	AT-rich interactive domain-containing protein 2		
YBX1	Nuclease-sensitive element-binding protein 1		
CNOT4	CCR4-NOT transcription complex subunit 4		
YBX2	Y-box-binding protein 2		
CHD8	Chromodomain-helicase-DNA-binding protein 8		
ASH2L	Set1/Ash2 histone methyltransferase complex subunit ASH2		
BCOR	BCL-6 corepressor		
EWSR1	RNA-binding protein EWS		
CHD4	Chromodomain-helicase-DNA-binding protein 4		
MLL2	Histone-lysine N-methyltransferase MLL2		
ASXL3	Putative Polycomb group protein ASXL3		
TAF4	Transcription initiation factor TFIID subunit 4		
RBBP4	Histone-binding protein RBBP4		
TAF6	Transcription initiation factor TFIID subunit 6		
POLR1A	DNA-directed RNA polymerase I subunit RPA1		
MED12	Mediator of RNA polymerase II transcription subunit 12		
CSDA	DNA-binding protein A		

### Contribution of KPNA2 to Protein Synthesis and Ribosomal RNA Transcription

**Figure 4:**
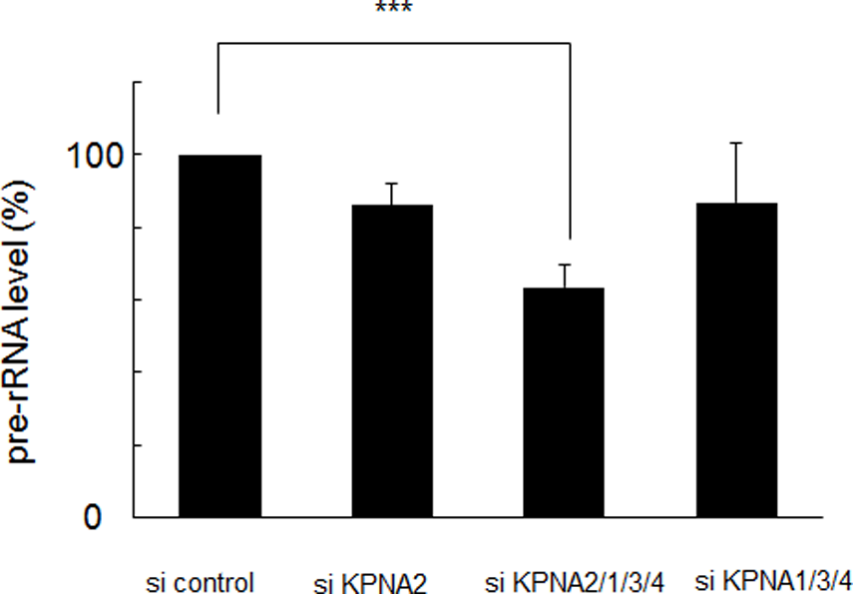
Suppression of ribosomal RNA synthesis by combined KPNA knockdown. Under starvation conditions (0.1% fetal bovine serum), siRNA-mediated knockdown of KPNA2, 1, 3, and 4 significantly suppressed ribosomal RNA synthesis analyzed by reverse transcription-quantitative polymerase chain reaction (***p<0.01). The amount of pre-ribosomal RNA was reduced by about 37% after 72 h.

**Figure 5:**
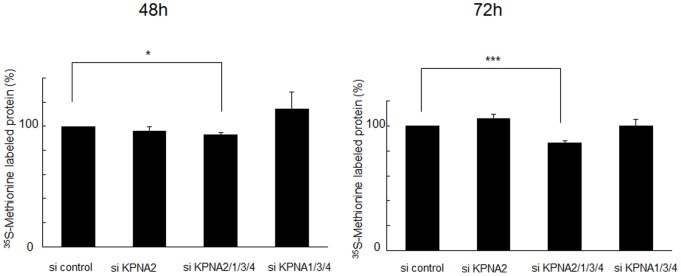
Suppression of protein synthesis by combined KPNA knockdown. Under starvation conditions (0.1% fetal bovine serum), siRNA-mediated knockdown of KPNA2, 1, 3, and 4 significantly suppressed protein synthesis after 48 h (*p<0.05) and 72 h (***p<0.01), as demonstrated by metabolic labeling with ^35^S-methionine.

**Figure 6:**
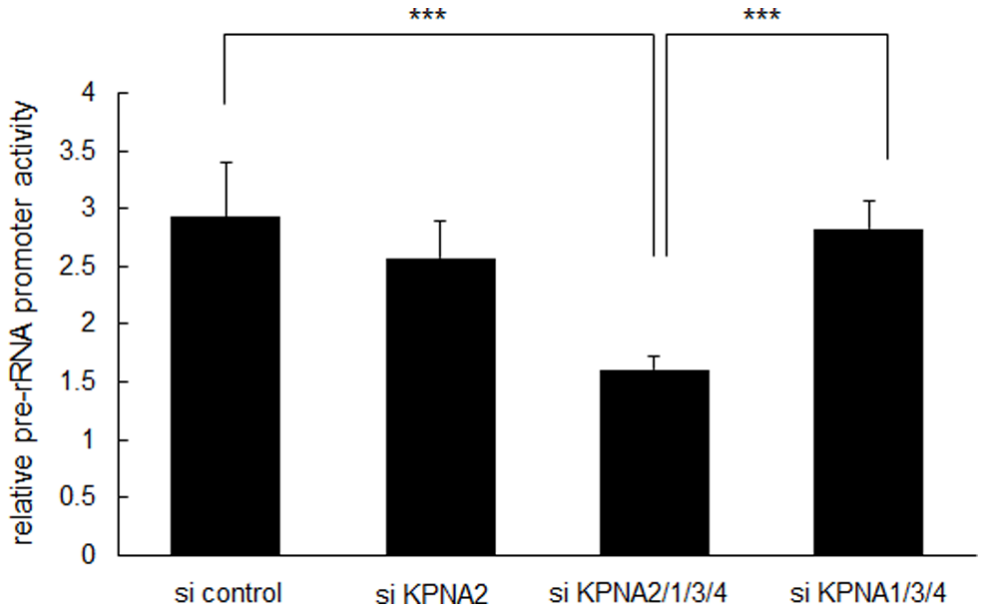
Suppression of the pre-ribosomal RNA promoter by combined KPNA knockdown. Under starvation conditions (0.1% fetal bovine serum), siRNA-mediated knockdown of KPNA2, 1, 3, and 4 significantly suppressed pre-rRNA promoter activity after 24 h (***p<0.01).

Because the nucleolus is specifically responsible for rRNA transcription and maintenance of gene expression/transcription and mRNA processing, we hypothesized that KPNA2 in the nucleoli may regulate rRNA transcription to maintain cell growth under starvation conditions. To test this hypothesis, the siRNA cocktail was again applied to knockdown KPNAs to observe the effect on pre-rRNA transcription in starved HaCaT keratinocytes. Knockdown of all KPNAs reduced pre-rRNA levels as measured by RT-qPCR. In the 72 h after treatment with the siRNA cocktail, pre-rRNA expression was reduced by about 40%. Subtraction of KPNA2 siRNA restored pre-rRNA expression. The other KPNAs did not contribute to pre-rRNA expression. Treatment with KPNA2 siRNA alone had no significant effect, suggesting a redundant mechanism with other KPNAs **(** **Figure 4** **)**. Protein synthesis in HaCaT keratinocytes was also reduced, corresponding to the suppression of pre-rRNA expression **(** **Figure 5** **)**. The pre-rRNA promoter was also suppressed by KPNA knockdown after 24 h (**Figure 6**). Fluorescence-activated cell sorting of HaCaT cells before and after KPNA knockdown showed no significant change in the cell cycle pattern (data not shown). These data suggest KPNA2 might positively regulate rRNA transcription in the nucleolus, maintaining cell growth by ensuring transcription and translation directly or indirectly.

## Discussion

In this study, KPNA2 was overexpressed in proliferating disorders of the skin and interacted with many kinds of proteins that control transcription and gene expression directly and indirectly. This was the first report to show that KPNA2 is essential for cell growth in terms of rRNA and protein synthesis under starvation conditions.

KPNA2 overexpression in several skin malignancies is associated with varying prognoses. In the basal cells of psoriasis, KPNA2 expression was diffusely up-regulated in comparison to atopic dermatitis. Thus, KPNA2 expression might be induced in cells in which proliferation has been activated. Comparing Bowen’s disease and actinic keratosis, which are known as SCC *in situ*, KPNA2 was remarkably and diffusely overexpressed. KPNA2 may therefore be a tumor marker with utility as a prognostic factor of proliferative activity in skin malignancies, although we have insufficient sample sizes to determine significance. Previous reports have demonstrated KPNA2 overexpression in various tumors cells *in vitro* and *in vivo*; elevated KPNA2 and KPNB1 expression in cancer cells correlates with altered transcriptional regulation associated with deregulated E2F/Rb activities [26]. Some studies have indicated that higher KPNA2 expression in tumor cell nuclei shortens patient survival, although the mechanisms and precise roles of KPNA2 in the tumor cells remained unclear [16], [17]. Researchers also hypothesized that KPNA2-mediated nuclear transport of proteins necessary for maintaining cell proliferation, such as transcription factors, promote tumor cell growth. In this context, KPNA2 was shown to interact with NBS1 (Nijmegen breakage syndrome 1), a key regulator of the MRE11/RAD50/NBS1 complex. NBS1 promotes tumorigenesis by binding and activating the phosphatidylinositol 3-kinase/AKT pathway [27]. Interestingly, siRNA-medicated KPNA2 knockdown studies revealed a different cellular response to KPNA2 inhibition in prostate and cervical cancer cell lines. In prostate cell line PC3, proliferation and viability were significantly reduced when KPNA2 expression was inhibited, whereas there was no significant change in a cervical cancer cell line. This difference could be due to tissue-specific tumor etiologies [13], [17].

In this study, we characterized KPNA2-binding proteins *in situ* in immortalized HaCaT keratinocytes. In silico gene ontology indicated a significant relationship between KPNA2 binding proteins and mRNA processing, ribonucleoprotein complex biogenesis, chromatin modification, and transcription. KPNA2 interacted with various ribosomal proteins and heterogeneous nuclear ribonucleoproteins directly or indirectly and was located in the nucleolus, the site of pre-rRNA transcription and processing and ribosome assembly. rRNA synthesis, the first event in ribosome synthesis, is a fundamental determinant of a cell’s capacity to grow and proliferate. Ribosomal RNA genes (rDNAs) are transcribed with high efficiency and the complex regulation of rRNA synthesis is responsive to general metabolism and specific environmental challenges [20], [28]. Serum starvation is also a well-established approach to inducing a broad range of cellular stress. TAP analysis revealed that KPNA2 associates with numerous ribosomal RNA synthesis-related proteins including RNA polymerase I subunit, rRNA methyl transferase, rRNA subunit biogenesis protein, and rRNA processing proteins. Furthermore, KPNA2 accumulates in the nucleolus and contributes to rRNA transcription *in vitro*. These lines of evidence suggest KPNA2 may serve important roles as a canonical nuclear transporter and to ensure rRNA biogenesis in proliferating cells. In this context, enhanced KPNA2 expression in malignant and inflammatory keratinocytes may positively regulate their proliferating capacity by supporting rRNA synthesis, which is indispensable. In malignant cells, the poor prognosis indicated by nuclear KPNA2 accumulation may be associated with KPNA2 retention in response to cellular stress and increasing rRNA synthesis or changing gene expression.

KPNA subtypes exhibit different abilities to interact with specific NLS-containing cargos and in various expression patterns in cells and tissues. KPNA2 is highly expressed in undifferentiated embryonic stem cells and down-regulated during neural differentiation, indicating that proper expression of KPNA2 is required for embryonic stem cells to maintain their undifferentiated state [29]. KPNs are also complementary because they are indispensable for cellular proliferation and differentiation. We previously examined the effect of KPNA2 siRNA subtraction on RNA expression in normal human keratinocytes by microarray analysis [4]; however, there was no increases of more than 2 fold in any other KPNs including KPNA1, 3, 4, and KPNB1 (data not shown).

In this study, KPNA2 was essential for cell growth related to rRNA and protein synthesis under starvation conditions; however, there was no significant change when only KPNA2 was knocked down. Combined knockdown of KPNA2, 1, 3, and 4 was needed to suppress cell growth and KPNA2 was indispensable. Even under these conditions, growth suppression was gradual and mild over 120 h. Furthermore, combined knockdown of KPNAs mildly suppressed the synthesis of rRNA and proteins after 72 h. These results indicated that KPNAs might play complementary roles with sufficient reserves.

Further studies are needed to clarify the additional function of KPNA2 in cell proliferation, which would be a focus for a new treatment to regulate KPNA2.
